# Fellow-Workers and the Roll of Honour

**Published:** 1915-03-20

**Authors:** 


### ROUND THE WORLD.

[The Editor invites Early Information and Contributions Marked "Fellow-Workers."]

### Fellow-Workers and the Roll of Honour.

Major T. E. Kaiser, a prominent resident of Oshawa, Ontario, has received his present honorary rank in the Canadian Militia as a reward for his patriotism and keen work in the service of the Empire. He has been most energetic not only in regard to recruiting and the Patriotic Fund, but also in regard to the erection of an armoury at Oshawa.

The to us primitive conditions under which medical work has to be carried out in Serbia have already resulted in the death of one of the British medical officers who have so gallantly responded to the call of that harassed country, and it has been a lady doctor, Miss Elizabeth Ness MacBean Ross, who has thus succumbed. A student of Glasgow University, Miss Ross qualified by taking the M.B., Ch.B. there in 1901, and subsequently took out courses of clinical instruction in Ireland and on the Continent. She had given special attention to the study of tropical diseases, her knowledge of which would doubtless have been of great value to her in the fever-stricken Serbian districts had not typhus fever overcome her at the outset of her work. Miss Ross had been inspector of midwifery for Durham county and resident medical officer at the Drumcondra Hospital, Dublin; in addition to this she had acted as surgeon to a steamship company and travelled a good deal in the East.

Mr. Donald John Armour, F.R.C.S. Eng., M.R.C.P. Lond., one of the surgeons to the King George Hospital, is on the staff of the West London Hospital and lecturer on surgery at the post-graduate college affiliated thereto, of which he is dean. Mr. Armour is a Toronto man and took the M.B. of the university there with honours before taking the conjoint qualifying diploma in London, where he studied at University College Hospital. He is also surgeon to the National Hospital for Paralysis and Epilepsy and consulting surgeon to the Blackheath and Charlton Hospital. In the course of a successful career Mr. Armour has been Hunterian professor, Jackson prize essayist, and Arris and Gale lecturer at the Royal College of Surgeons.

Mr. Joseph Ebenezer Adams, who has also been appointed surgeon to the King George Hospital, is an out-patient surgeon at St. Thomas's Hospital, senior demonstrator of anatomy in the medical school, and surgeon to the East London Hospital for Children. An M.S. Lond., F.R.C.S. Eng., he won the Beaney scholarship in surgery at St. Thomas's, and first-class honours in anatomy at the intermediate M.B. Lond. A past Hunterian professor of the Royal College of Surgeons, he was formerly resident assistant surgeon, surgical registrar, and house surgeon at his hospital.

One of the most remarkable features of the war has been the application of the motor car to ambulance work; not only have we motor ambulances and laboratories nowadays, but a motor operating theatre has lately been constructed. Mr. George Herbert Colt, the designer of this novel life-saving appliance, of which a good deal more will be heard in the near future, is assistant surgeon to the Royal Aberdeen Infirmary and assistant to the professor of surgery in Aberdeen University. He has for some time been interested in ambulance work, and his name appears in the list of captains attached to the 1st Scottish Territorial Hospital. An M.B., B.C. Cantab., F.R.C.S. Eng., Captain Colt went to "Bart.'s" from Sidney Sussex College, Cambridge, and there, after qualification, held the posts of house surgeon and resident anæsthetist. Before settling down in practice he was for a time senior house surgeon to the Derby Royal Infirmary. It is to be noted that he described a portable theatre in the *British Journal of Surgery* some two years ago.

Captain Harry Morell, of the Canadian Army Medical Corps, has been making important observations in the matter of anti-typhoid inoculation. Reporting recently to the *British Medical Journal* on his experiences, Captain Morell related that when the first Canadian Expeditionary Force was dealt with 27,000 men accepted this important prophylactic measure, their acceptance calling for some 57,000 injections. In spite of the comparative rapidity with which these inoculations had to be carried out, no instance of serious complications occurred.

Three other well-known pathologists now attached to the military forces who have been also investigating anti-typhoid inoculation lately are Dr. Georges Dreyer, Dr. E. W. Ainley Walker, and Dr. Alexander Gibson. These three investigators have reported (vide *Lancet*, February 13, 1915) also strongly in favour of prophylactic paratyphoid inoculations under certain conditions of intestinal trouble likely to be confused with true enteric fever, but really due to the *Bacillus paratyphosus*.

Dr. Georges Dreyer is Professor of Pathology at Oxford University, and now also honorary consulting pathologist to the 3rd Southern General Hospital. A student of the University of Copenhagen, where he took his M.D. degree, Professor Dreyer is also a Fellow of Lincoln College, Oxford, and holds the M.A. degree of that university. His original work includes important observations on immunity, experimental pathology, and the biochemical effect of light rays.

Dr. E. W. Ainley Walker, M.A., M.D. Oxon., now honorary assistant pathologist to the 3rd Southern General Hospital, is lecturer on pathology at Oxford, and a well-known writer on pathological matters. He did his clinical work at the London Hospital, where he won the prize entrance scholarship in 1896 and the Sutton scholarship in 1898, subsequently becoming a Radcliffe Travelling Fellow. Before devoting his attention entirely to the particular branch of medical science in which he has done such brilliant work, Dr. Ainley Walker was house physician at the London Hospital. His past appointments also include those of examiner in pathology at Cambridge University, and the Gordon lectureship in experimental pathology and directorship of the pathological department at Guy's Hospital.

Captain Alexander George Gibson, R.A.M.C. (T.), pathologist to the 3rd Southern General Hospital, is a prominent member of Oxford University, where he holds the post of lecturer in morbid anatomy and lecturer and examiner in pathology, and is also assistant pathologist to the Radcliffe Infirmary and County Hospital at Oxford. A St. Thomas's Hospital man, he qualified by taking the M.B., B.Ch. Oxon., subsequently obtaining the M.D. degree and being elected an F.R.C.P. Lond.

